# Exploring the nitrogen reservoir of biodegradable household garbage and its potential in replacing synthetic nitrogen fertilizers in China

**DOI:** 10.7717/peerj.12621

**Published:** 2022-01-18

**Authors:** Lan Wang, Tianyu Qin, Jianshe Zhao, Yicheng Zhang, Zhiyuan Wu, Xiaohui Cui, Gaifang Zhou, Caihong Li, Liyue Guo, Gaoming Jiang

**Affiliations:** 1 State Key Laboratory of Vegetation and Environmental Change, Institute of Botany, the Chinese 17 Academy of Sciences, Beijing, China; 2 College of Resources and Environment, University of Chinese Academy of Sciences, Beijing, China; 3 Henan Zhongyuan Organic Agriculture Research Institute Co., Ltd., Zhengzhou, China

**Keywords:** Household garbage, Food waste, Biomass nitrogen reservoir, Agricultural sustainable development

## Abstract

Biodegradable household garbage contains a large amount of nitrogen, which could be used as organic fertilizer to produce organic food and significantly reduce synthetic nitrogen fertilizers. There is limited information on how large the nitrogen reservoir of biodegradable household garbage is in a certain country or region. Here we took China as a case, analyzed the amount of biodegradable household garbage resources and their nitrogen reservoirs. It was noted that the biodegradable household garbage mainly included food waste, waste paper and wood chips, with the amount being 31.56, 29.55, and 6.45 × 10^6^ t·a^−1^, respectively. Accordingly, the nitrogen reservoirs were 65.31 × 10^4^, 6.80 × 10^4^, and 3.81 × 10^4^ t·a^−1^ in China. The nitrogen reservoir of food waste accounted for 86% of the total nitrogen reservoir of biodegradable household garbage, which was equivalent to 11% of the amount of actual absorption for synthetic nitrogen fertilizers (6.20 × 10^6^ t·a^−1^) by agriculture plants in China. Our findings provided a scientific basis for the classification and utilization of biodegradable household garbage.

## Introduction

Modern agriculture is mainly characterized with large utilization of synthetic chemical substances such as fertilizers, pesticides, and herbicides. Although the crop yields have been increased by chemical substances, the long-term application of those chemicals has caused serious adverse effects on soil, air, water, food, even human health. Soil acidification (Guo et al., 2010; Sutton et al., 2011), greenhouse gas emission (Parihar et al., 2018), water eutrophication (Hansen, Termansen & Hasler, 2019) and pesticide residues (Urso & Gilbertson, 2018) in agricultural system have been frequently reported. As a result, the quality of food, the nutritional contents (Yu et al., 2018), and the incomes of agriculture have been largely decreased (Stuart & Houser, 2018). To a certain extent, cheaper food lead to more food waste. For instance, today, food mixed with garbage become the main components of modern urban and rural household garbages.

In the world, a large amount of household garbage is produced every day, which causes serious environmental pollution, and cost a lot of lands and money to handle (Du et al., 2018). According to the statistics of the World Bank (2018), the amount of household garbage generated worldwide reached to 2.01 × 10^9^ t·a^−1^ in 2016. In China, 4 × 10^8^ t·a^−1^ household garbages (fresh weight) have been produced, while the disposal rate was far behind its rate of generation (Wu et al., 2018).

The accumulation of household garbage has resulted in increasing environmental pollution, affecting the lives of residents and harming human health (Hiramatsu et al., 2009; Rao & Rathod, 2019). Actually, the household garbage has an abundance of nutrients, including organic matter (39.05%), nitrogen (1.02%), phosphorus (0.50%) and potassium (1.42%) (Han et al., 2019). Nitrogen, an essential nutrient for plants growth, is the key element in the agricultural ecosystem (Sharma & Bali, 2018), particularly in poor soils which need more exogenous nitrogen as an essential nutrient (Zeng et al., 2020). Globally, 1.50–2.00 × 10^8^ t·a^−1^ mineral nitrogen is required to produce grains, feed animals and be used as industrial products (Aulakh et al., 2017). Nowadays, the demand for nitrogen is mainly met by applying synthesized nitrogen fertilizers (Lu & Tian, 2017). If the biodegradable household garbage was utilized as organic fertilizer, we would reduce the application amount of synthesized nitrogen at a certain level.

The household garbage comes from both urban and rural waste. Urban household garbage, known as municipal solid waste, includes food waste, recyclables, hazardous waste and others. However, the largest proportion in municipal solid waste is food waste, accounting to 61.2% of the total (Gu et al., 2017). The main components of rural household garbage in China include inert waste, food waste, glass and paper (Han et al., 2019). Regardless of urban or rural household garbage, biodegradable food waste accounts for a large proportion (Gallipoli et al., 2020), with the main components being carbohydrate polymers (starch, cellulose, hemicellulose), proteins, organic acids, lignin, lipids, *etc*. Those biological materials can be decomposed into reducing sugars, free amino acids, phosphates and nitrates under the action of microbial hydrolysis which could be absorbed by agriculture plants (Xing et al., 2019). In addition, food waste has low content of salts and heavy metals, which might be directly used as organic fertilizer (Xiong, 2015).

Among 94 countries in the world, China takes a leading position in the amount of food waste, followed by the United States and South Korea (Zhang et al., 2018). However, intensive researches have just forcused on the technology innovation, management methods and bioenergy utilization potentials of food waste, in some countries such as the United States (Breunig et al., 2017), South Korea (Seo et al., 2019), and Ireland (Dennehy et al., 2017), Turkey (Guven, Wang & Eriksson, 2019), *etc*. In many European countries, there were more reports on food waste composting technology (Ghinea et al., 2019), and food waste collection methods (Cecchi & Cavinato, 2019). Although the UK had the most detailed, directly measured household food waste data, only the nutrients and energy content had been calculated (Cooper et al., 2018). Some investigators from Italy (Stathopoulos, 2017; Giordano, Alboni & Falasconi, 2019) and Canada (von Massow et al., 2019; Heidari et al., 2020) analyzed the main behaviors and attitudes based on the amount of food waste. Nevertheless, there was no statistics on the overall nitrogen reservoir. It is still not clear the resources and nitrogen reservoir of the biodegradable components of household garbage in the developing countries like China. The potential of replacing chemical fertilizers has been seldom reported, so the relevant scientific research is urgently needed.

The scientific hypothesis of this paper was that the biodegradable household garbage contained a large amount of nitrogen resources that could significantly replace the synthetic nitrogen fertilizers to develop organic agriculture. The treatment of biodegradable household garbage and the development of organic agriculture could be perfectly unified with the help of farmland. This study tried to use the statistical data to reveal: (1) The biodegradable components and resources in urban and rural household garbage together with the nitrogen reservoir in China; (2) the potential of biodegradable household garbage in replacing chemical fertilizers. This study might provide scientific basis for both the utilization of household garbage resources and the health development of organic agriculture.

## Materials and Methods

### Data sources

The biodegradable household garbage was divided into urban and rural one. According to the composition and characteristics, the biodegradable garbage was further divided into three categories, *i.e*., food waste, waste paper and wood chips (Li et al., 2019). Data were collected as previously described in Cui date (Cui et al., 2021). Specifically the original data mainly came from: China Statistical Yearbook (2011–2020; http://www.stats.gov.cn/tjsj/ndsj/2019/indexch.htm) and China Statistical Yearbook on Urban and Rural Construction (2011–2020; http://www.mohurd.gov.cn/xytj/tjzljsxytjgb/jstjnj/index.html). The rest of related information was obtained by searching “household garbage”, “biodegradable household garbage”, and “food waste” through “Web of Science” and “Chinese National Knowledge Infrastructure (CNKI)”. Because of the special statistical system and the situation of China, all data reported in this paper were included except Hong Kong, Macau and Taiwan.

### Food waste resources and the nitrogen reservoir

The total amount of food waste resources contained urban and rural one. The former was calculated by multiplying urban household garbage by the proportion of urban food waste in urban household garbage. The latter was calculated by multiplying the rural population by the average daily waste generation *per capita*, the number of days per year and the ratio of rural food waste to rural household garbage. The nitrogen reservoir of food waste was calculated by multiplying the total amount of food waste by its average nitrogen content.

(1) $$\eqalign{ \rm TF &= (\rm UF + RF)\times (1 - {\it w}1) = (UG\times a1+RG \times b1) \times (1 - {\it w}1) \cr&= (\rm UG \times a1 + RP \times DG \times {\it D} \times b1) \times (1 - {\it w}1)}$$

(2) $$\rm TN1= TF \times \alpha 1$$

where TF is the total output of urban and rural food waste each year; UF is the the output of urban food waste; RF is the output of rural food waste; UG is the output of urban household garbage; RG is the output of rural household garbage; RP is the number of rural population; DG is the *per capita* daily garbage production; a1 is the proportion of urban food waste in urban household garbage (61.20%) (Gu et al., 2017); b1 is the proportion of rural food waste in rural household garbage (33.7%) (Wu et al., 2018); *D* is the total days of the year; *w*1 is water content (82%) (Gallipoli et al., 2020); TN1 is the total urban and rural food waste nitrogen reservoir; *α*1 is average nitrogen content of food waste (2.07%) (Adhikari et al., 2009; Yang et al., 2013; Zhang et al., 2016).

### Waste paper resources and the nitrogen reservoir

The total amount of waste paper resources was composed of urban waste paper and rural waste one. The former was calculated by multiplying urban household garbage by the proportion of urban waste paper in urban household garbage. The latter was calculated by multiplying the rural population by the average daily waste generation *per capita*, the number of days per year and the ratio of rural waste paper to rural household garbage. The nitrogen reservoir of waste paper was calculated by multiplying the total amount of waste paper by its average nitrogen content.

(3) $$\eqalign{ \rm TP &= (\rm UP + RP) \times (1 - {\it w}2)= (UG \times a2+ RG \times b2) \times (1 - {\it w}2) \cr&= (\rm UG \times a2 + RP \times DG \times {\it D} \times b2) \times (1 - {\it w}2)}$$

(4) $$\rm TN2 = TP \times \alpha 2$$

where TP is the total output of urban and rural waste paper each year; UP is the the output of urban waste paper; RP is the output of rural waste paper; UG is the output of urban household garbage; RG is the output of rural household garbage; RP is the number of rural population; DG is the *per capita* daily garbage production; a2 is the proportion of urban waste paper in urban household garbage (9.6%) (Gu et al., 2017); b2 is the proportion of rural waste paper in rural household garbage (10.75%) (Wu et al., 2018); *D* is the total days of the year; *w*2 is water content (7.35%) (Ding et al., 2013); TN2 is the total urban and rural waste paper nitrogen reservoir; *α*2 is average nitrogen content of waste paper (0.23%) (Ding et al., 2013).

### Wood chip resources and the nitrogen reservoir

The total amount of wood chips resources was composed of urban wood chips and rural ones. The former was calculated by multiplying urban household garbage by the proportion of urban wood chips in urban household garbage. The latter was calculated by multiplying the rural population by the average daily waste generation *per capita*, the number of days per year and the ratio of rural wood chips to rural household garbage. The nitrogen reservoir of wood chips was calculated by multiplying the total amount of wood chips by their average nitrogen content.

(5) $$\eqalign{ \rm TW &= (\rm UW + RW) \times (1 - {\it w}3)= (UG \times a3+ RG \times b3) \times (1 - {\it w}3) \cr&= (\rm UG \times a3 + RP \times DG \times D \times b3) \times (1 - {\it w}3)}$$

(6) $$\rm TN3 = TW \times \alpha 3$$

where TW is the total output of urban and rural wood chips each year; UW is the the output of urban wood chips; RW is the output of rural wood chips; UG is the output of urban household garbage; RG is the output of rural household garbage; RP is the number of rural population; DG is the *per capita* daily garbage production; a3 is the proportion of urban wood chips in urban household garbage (1.8%) (Gu et al., 2017); b3 is the proportion of rural wood chips in rural household garbage (3.23%) (Wu et al., 2018); *D* is the total days of the year; *w*3 is water content (7.24%) (Zhou, Selvam & Wong, 2018; TN3 is the total urban and rural wood chips nitrogen reservoir; *α*3 is average nitrogen content of wood chips (0.59%) (Zhou, Selvam & Wong, 2018).

### Total biodegradable household garbage nitrogen reservoir

The total nitrogen reservoir of urban and rural biodegradable household garbage included urban and rural food waste nitrogen reservoir, waste paper nitrogen reservoir, and wood chips nitrogen reservoir, which was calculated as followings:

(7) $$\rm TN= TN1+TN2+ TN3$$

TN is the total urban and rural biodegradable household garbage nitrogen reservoir; TN1 is the total urban and rural food waste nitrogen reservoir; TN2 is the total urban and rural waste paper nitrogen reservoir; TN3 is the total urban and rural wood chips nitrogen reservoir.

### Statistical analysis

Microsoft Excel 2007 was applied to process the data. SPSS 20.0 (SPSS Inc., Chicago, IL, USA) was applied to analyse the data. Figures were generated using SigmaPlot 12.5 (Systat Software Inc., San Jose, CA, USA).

## Results

### Food waste resource and its nitrogen reservoir

**Figure 1:**
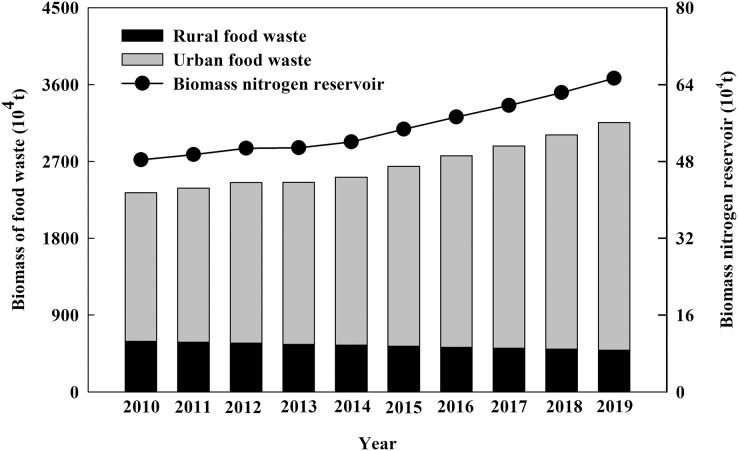
The biomass and nitrogen reservoir of urban and rural food waste in China from 2010 to 2019.

The total amount of urban and rural household garbage resources in China dated from 2010 to 2019 was shown in Table 1. According to Eq. (1), the amount of urban and rural food waste (dry weight) in China had been increasing year by year, reaching to 3.16 × 10^7^ t·a^−1^ in 2019. As well, the amount of urban food waste continued to grow, reaching to 2.67 × 10^7^ t·a^−1^ in 2019. Compared with 2010, the urban food waste increased by 53%, accounting 88% of the total food waste. However, as more and more rural people flooded into cities, the amount of rural food waste decreased, from 5.94 × 10^6^ t·a^−1^ in 2010 to 4.89 × 10^6^ t·a^−1^ in 2019 (Fig. 1). According to Eq. (2), the nitrogen reservoir of food waste had been also increasing yearly, reaching to 6.51 × 10^5^ t·a^−1^ in 2019 (Fig. 1).

**Table 1:** Urban and rural household garbage resources in China from 2010 to 2019 (fresh weight).

Household garbage		2010	2011	2012	2013	2014	2015	2016	2017	2018	2019
Urban	Production (10^4^t)	15,438	15,734	15,805	16,395	17,081	17,239	17,860	19,142	20,362	21,521
Rural	Population(10^4^)	67,113	65,656	64,222	62,961	61,866	60,346	58,973	57,661	56,401	55,162
	*Per capita* daily (kg/d)	0.4	0.4	0.4	0.4	0.4	0.4	0.4	0.4	0.4	0.4
	Days	365	365	366	365	365	365	366	365	365	365
	Production (10^4^t)	9,798	9,586	9,402	9,192	9,032	8,811	8,634	8,419	8,235	8,054
Total	Production (10^4^t)	25,603	25,981	26,483	26,431	26,892	27,953	28,996	29,940	31,037	32,260

### Waste paper resource and its nitrogen reservoir

**Figure 2:**
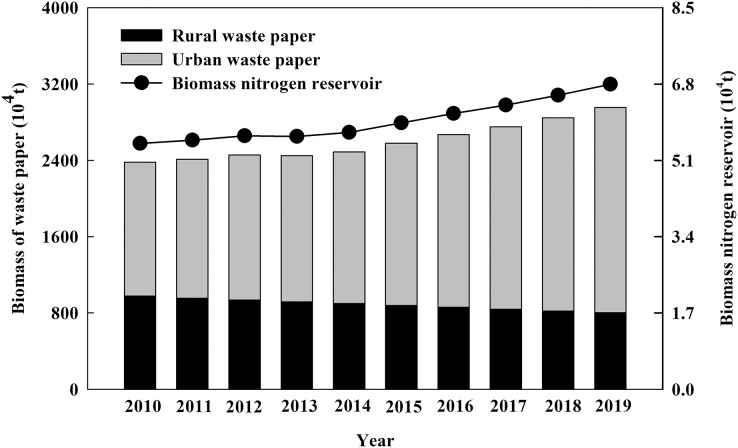
The biomass and nitrogen reservoir of urban and rural waste paper in China from 2010 to 2019.

It was noted that the amount of urban waste paper resources elevated from 1.41 × 10^7^ t·a^−1^ in 2010 to 2.15 × 10^7^ t·a^−1^ in 2019 after calculated by Eq. (3). The amount of rural waste paper resource decreased from 9.76 × 10^6^ t·a^−1^ to 8.02 × 10^6^ t·a^−1^. The total amount of urban and rural waste paper resources increased year by year, reaching to 2.96 × 10^7^ t·a^−1^ in 2019. According to Eq. (4), the nitrogen reservoir of waste paper increased with the increase of waste paper resource, reaching to 6.80 × 10^4^ t·a^−1^ in 2019 (Fig. 2).

### Wood chips resource and its nitrogen reservoir

**Figure 3:**
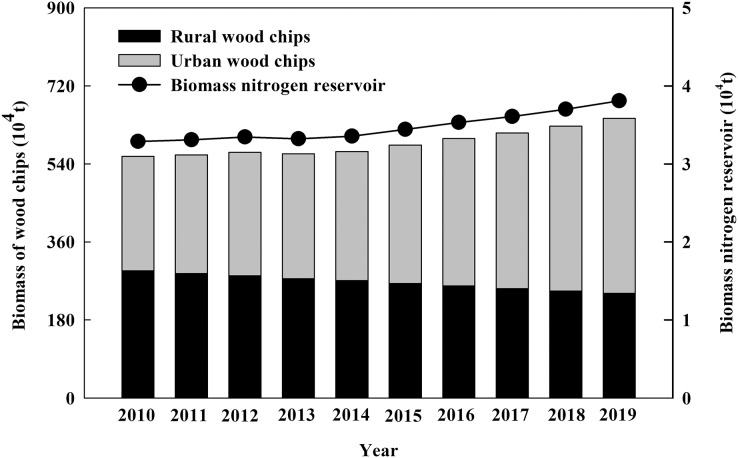
The biomass and nitrogen reservoir of urban and rural wood chips in China from 2010 to 2019.

From 2010 to 2019, the amount of urban wood chips increased from 2.64 × 10^6^ t·a^−1^ to 4.04 × 10^6^ t·a^−1^ after calculated by Eq. (5). The amount of rural wood chips decreased from 2.94 × 10^6^ t·a^−1^ to 2.41 × 10^6^ t·a^−1^. The total amount of wood chips in urban and rural increased yearly, reaching to 6.45 × 10^6^ t·a^−1^ in 2019. According to Eq. (6), the wood chip nitrogen reservoir increased to 3.81 × 10^4^ t·a^−1^ in 2019 (Fig. 3).

### The total nitrogen reservoir of biodegradable household garbage and its components

**Figure 4:**
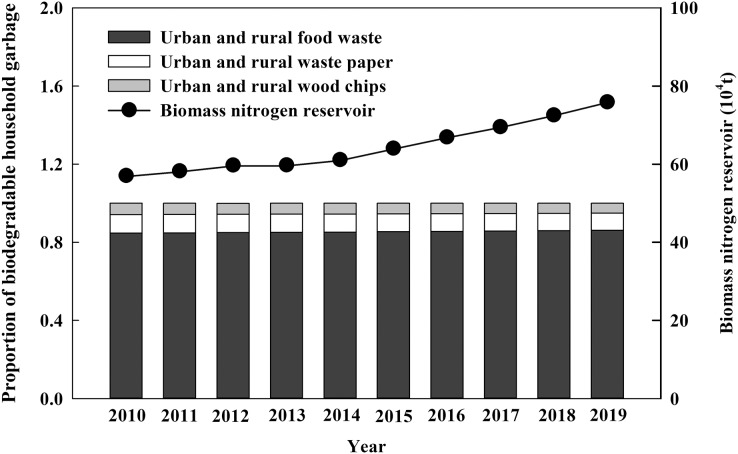
Changes of nitrogen reservoir and the proportion of biodegradable household garbage from 2010 to 2019 in China.

It was found that the total nitrogen reservoir of urban and rural biodegradable household garbage in China had been increasing since 2010. In 2019, the nitrogen reservoir was 75.92 × 10^4^ t·a^−1^, increased by 33% compared with that of 2010 (Fig. 4). For the components, urban and rural biodegradable household garbage were composed of food waste, waste paper and wood chips, among which food waste accounted for 84–86%, waste paper 8–9% and wood chips 5–6%. The proportion of food waste had been increasing, while the proportion of waste paper and wood chips had been decreasing from 2010 to 2019 (Fig. 4).

### Yearly rates of changes in biodegradable household garbage resources

From 2010 to 2019, the yearly changes of urban biodegradable household garbage resources had increased while that of rural had decreased in China (Table 2). From 2014 to 2015, the yearly rate of increase in urban biodegradable household garbage resources was the maximum. From 2016 to 2017, however, the yearly rate of decrease in rural household gargage was the maximum.

**Table 2:** Urban increase and rural decrease yearly rates of biodegradable household garbage resources in China from 2010 to 2019.

Year	Biodegradable household garbage	
	Urban yearly increase rates (%)	Rural yearly decrease rates (%)
2010–2011	3.73	2.17
2011–2012	4.18	1.92
2012–2013	0.93	2.23
2013–2014	3.60	1.74
2014–2015	7.18	2.46
2015–2016	6.37	2.01
2016–2017	5.69	2.49
2017–2018	5.95	2.19
2018–2019	6.16	2.20

### *Per capita* biodegradable household garbage resources in seven regions of China

In 2019, the amount of urban and rural food waste, waste paper, wood chips and the *per capita* biodegradable household garbage resources in seven regions of China were shown in Table 3. The largest amount of biodegradable household garbage and the *per capita* biodegradable household garbage were from east and sourthern China, respectively, with the least being from northwest China.

**Table 3:** Urban and rural *per capita* biodegradable household garbage resources in seven regions of China in 2019.

		North	Northeast	East	Central	Southern	Southwest	Northwest
Urban	Food waste (10^4^t)	331.42	219.45	884.92	318.36	451.82	292.54	165.08
	Waste paper (10^4^t)	267.59	177.18	714.49	257.05	364.80	236.20	133.29
	Wood chips (10^4^t)	50.23	33.26	134.13	48.25	68.48	44.34	25.02
Rural	Food waste (10^4^t)	54.94	35.23	127.69	86.63	54.08	83.92	41.62
	Waste paper (10^4^t)	90.21	57.85	209.66	142.24	88.79	137.79	68.33
	Wood chips (10^4^t)	27.14	17.40	63.07	42.79	26.71	41.45	20.56
Total	Biodegradable household garbage (10^4^t)	821.53	540.37	2,133.95	895.34	1,054.68	836.24	453.89
	Population (10^4^)	17,577	10,794	41,423	22,485	17,426	20,331	10,349
	*Per capita* (kg a^−1^)	46.74	50.06	51.52	39.82	60.52	41.13	43.86

### The relationship between per capital GDP and per capital discharge of food waste

Among the biodegradable household garbage, food waste accounted for the largest proportion, which was the most promising renewable resource as organic fertilizer. In 2019, the amount of urban and rural food waste resources in different provinces of China were shown in Table 4 (Provincial scale). The top five provinces with the largest amount of food waste were from Guangdong, Jiangsu, Shandong, Zhejiang and Henan. As one of most developed provinces in China, Guangdong had the maximum food waste discharg of 3.98 × 10^6^ t·a^−1^, accounting for 13% of the total food waste of the country.

**Table 4:** Gross domestic product (GDP) and food waste resources in 2019 in China.

Province	Population (10^4^)	GDP (billion, $)	Urban and rural food waste production (10^4^t)	*Per capita* GDP ($)	*Per capita* food waste (kg)
Guangdong	11,521	1,648.74	397.92	14,310.74	34.54
Jiangsu	8,070	1,525.63	220.35	18,904.96	27.30
Shandong	10,070	1,088.24	231.16	10,806.75	22.96
Zhejiang	5,850	954.78	184.11	16,321.03	31.47
Henan	9,640	830.86	164.94	8,618.88	17.11
Sichuan	8,375	713.82	163.01	8,523.22	19.46
Hubei	5,927	701.76	128.43	11,840.05	21.67
Fujian	3,973	649.18	118.32	16,339.79	29.78
Hunan	6,918	608.71	111.63	8,798.93	16.14
Shanghai	2,428	584.26	85.21	24,063.43	35.09
Anhui	6,366	568.32	96.09	8,927.43	15.09
Beijing	2,154	541.63	113.95	25,145.31	52.90
Hebei	7,592	537.55	116.87	7,080.48	15.39
Shaanxi	3,876	394.96	83.76	10,189.89	21.61
Liaoning	4,352	381.43	120.84	8,764.48	27.77
Jiangxi	4,666	379.11	77.37	8,124.95	16.58
Chongqing	3,124	361.47	75.47	11,570.74	24.16
Yunnan	4,858	355.62	72.20	7,320.30	14.86
Guangxi	4,960	325.20	76.31	6,556.45	15.39
Inner Mongolia	2,540	263.57	51.70	10,376.77	20.35
Shanxi	3,729	260.73	68.48	6,991.95	18.36
Guizhou	3,623	256.78	56.52	7,087.50	15.60
Tianjin	1,562	215.98	35.36	13,827.14	22.64
Heilongjiang	3,751	208.45	70.67	5,557.18	18.84
Xinjiang	2,523	208.21	48.75	8,252.48	19.32
Jilin	2,691	179.57	63.16	6,672.98	23.47
Gansu	2,647	133.50	42.88	5,043.45	16.20
Hainan	945	81.29	31.66	8,602.12	33.50
Ningxia	695	57.40	16.85	8,258.99	24.24
Qinghai	608	45.42	14.45	7,470.39	23.77
Tibet	351	26.00	9.25	7,407.41	26.35

**Note:**

$1.0 = 6.5305 Chinese Yuan.

**Figure 5:**
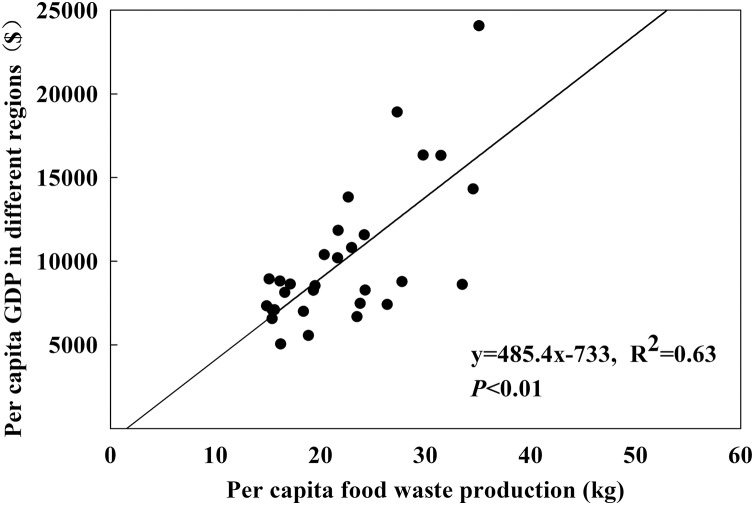
The relationship between per capital GDP and per capital discharge of food waste.

There was a pretty significant linear positive correlation between the per capital GDP and per capital discharge of food waste (*P* < 0.01) (Fig. 5). The per capital GDP values of different provinces were found to be mostly relevant with per capital food waste. The more GDP increased, the more foods were wasted, indicating that the fast development of economy was based on the huge waste of food resources which further led to environmental pollutions.

## Discussion

### Components and treatments of urban and rural degradable household garbage

Economic development, urbanization, and the living standards improvement of human being have led to a sharp increase in the amount of household garbage discharge, especially in developed countries (Breunig et al., 2017). To a certain extent, the discharge of household garbage is restricted by socio-economic conditions (Huang et al., 2013) and geographical locations (Han et al., 2015). Unfortunately, the developing countries have been following the footsteps of developed countries, with their garbage waste being increased rapidly (Rai et al., 2019). Most of cities in the world have struggled to “Garbage siege”. Unlike urban residents, rural residents have maintained a high utilization rate of those materials (Ma, Hipel & Hanson, 2018). In 2019, the total amount of urban and rural household garbage reached to 3.23 × 10^8^ t·a^−1^ in China (Table 1).

At present, the methods for treating urban household garbage mainly include incineration, landfill, composting, *etc*. (Zhang, 2019). In the rural China, however, there is still a shortage of appropriate infrastructure and solid waste management (Hiramatsu et al., 2009). Most rural household garbages are randomly discarded without any treatments, resulting in increase of environmental pollution and damage of human health (Cao, Xu & Liu, 2018).

Implementing household garbage classification which follows the principles of reduction, recycling, and harmlessness, is believed to be an effective approach to improve the urban and rural environments and promote resource recycling (Shi et al., 2020). However, it achieved limited effect, dispite of enough mobilization has been done by the government, as urban and rural residents always believe that garbage disposal is the government’s business not theirs. According to the compositions and characteristics of household garbage, urban and rural degradable household garbages are divided into food waste, waste paper and wood chips (Li et al., 2019). We here found these resources in China were respectively 31.56, 29.55 and 6.45 × 10^6^ t·a^−1^. In 2019, the total amount of the biodegradable household garbage was 67.4 × 10^6^ t·a^−1^ in China (Table 3). The urban biodegradable household garbage was on the rise over time (Table 2).

Urban and rural wood chips, as a kind of biomass, can be used as adsorbents for treating waste water, which reduces waste water treatment costs while increases environmental benefits (Li, 2010). Waste wood chips could be also converted into gaseous or liquid fuel, chemical raw materials and other products through thermochemical, chemical, and biological methods (Xu et al., 2018). Waste paper, however, is an ideal recyclable renewable resource that can be used for paper-making, wood production, or terated as various functional materials (Liu, 2016). In order to increase the utilization rate of paper, those waste papers are normally reused, even recycled, which could reduce the amount of felling of trees and obtain more ecological benefits (Liu, 2018). Therefore, the utilization of waste wood chips and waste paper follows a mode of circular economy. The application of such waste in the environmental protection industry should be continuously more strengthened rather than be used as fertilizers.

Food waste is the main component of urban and rural household garbage, which is also one of the most promising renewable resources (Cecchi & Cavinato, 2019). In the U.S. wastes about 63 × 10^6^ t·a^−1^ of food wasted (Dusoruth & Peterson, 2020), South Korea watstes about 5 × 10^6^ t·a^−1^ food (Lee et al., 2019), in Europe approximately 10.0 × 10^7^ t·a^−1^ of food are wasted (Zhang et al., 2018). Food waste reached to 3.16 × 10^7^ t·a^−1^ in 2019 in China (dry weight). Much of the food waste is a result of urbanization, for instance, urban food waste in China was as large as 2.67 × 10^7^ t·a^−1^, while rural food waste was only 4.89 × 10^6^ t·a^−1^ in the year of 2019 (Fig. 1). Food waste can be treated by landfill, incineration and composting (Han et al., 2015). However, landfill is likely to bring about greenhouse gases emissions, generation of large amount of leachate, and finally environmental pollution (Ma & Liu, 2019). Although incineration could reduce the volume of food waste, it needs a high demand for energy and might produce harmful substances and greenhouse gasses (Liu et al., 2019).

Aerobic composting is a relatively environmentally friend technology for food waste treatment, as foods contain high concentrations of easily degradable organic substances and nutrients which are easily to be decomposed (Hou et al., 2017). Studies have found that food waste composted with Chinese medicinal herbal residues (Zhou, Selvam & Wong, 2018), green waste (Williams et al., 2019), sugarcane leaves (Shan et al., 2019), cattle manure (Xing et al., 2019), pig manure (Dennehy et al., 2017; Jiang et al., 2018), chicken manure (Saad, Rahman & Yusoff, 2019) could be processed to form biological fertilizers, which would increase organic matters in the soil and improve the soil structure (Wang & Zeng, 2018). Therefore, the degradable food waste, especially rural food waste could be used on-site as fertilizers for organic crop production, which can largely reduce transportation and treatment costs and increase farmers’ income as well.

### Nitrogen reservoir of biodegradable household garbage in different components

In China, food waste accounted for the largest proportion (84–86%) of the nitrogen reservoir of biodegradable household garbage, followed by waste paper and wood chips. Along with time, the proportion of food waste increased, while waste paper and wood chips declined (Fig. 4). In 2019, we found that the nitrogen reservoir of food waste, waste paper and wood chips in China were 65.31 × 10^4^, 6.80 × 10^4^, and 3.81 × 10^4^ t·a^−1^, respectively.

The nitrogen in food waste was mainly organic one, which was found in various molecular forms, such as protein, amino acid, and nucleic acid, *etc*. (Wang & Zeng, 2018). It was reported that the protein content of food waste was 20% (Waqas et al., 2019) and NH_4_^+^–N was 2,800 mg kg^−1^ (Rigby & Smith, 2013). Nitrate is the main form of nitrogen absorption and utilization by most crops in cultivated soils (Andrews, Raven & Lea, 2013). So, as a source of biomass nitrogen, food waste can be used in organic agriculture to improve soil quality and promote agricultural development.

### Potential of biodegradable household garbage of replacing synthetic nitrogen fertilizers

Among the biodegradable household garbage, wood chips and waste paper could be recycled, which play more important roles in the environmental protection industry rather than used as fertilizers. Therefore, this article did not consider them as organic fertilizers. During fermentation process, various components in food waste are converted into stable humus-like substances and rapidly available nutrients, which can be quickly hydrolyzed (Bi et al., 2019), or directly absorbed by plants (Hou et al., 2017). Those materials could also improve nutrition levels of the soil (Du et al., 2018) and could be generally beneficial to soil microbial communities (Liu et al., 2020) which are closely related to soil fertility. Soil microorganisms provide a variety of services for agriculture ecosystem, such as cycling of nutrient elements, degradation of pesticides, suppression of plant diseases, and promotion of plant growth (Ding et al., 2019). In food waste, the dissolved organic matter is very active, which directly provides energy for microbes (Shan et al., 2019). Therefore, food waste is regarded as an ideal and cheap raw material for the production of biological fertilizers (Ma & Liu, 2019). It was documented that the rapid humification of food waste prepared as a soil conditioner could significantly improve the total organic carbon content in orchard soils (Jia et al., 2019). Some also found that food waste culture medium could replace inorganic culture medium as a nutrient supplement to cultivate chlorella and improve nutrient utilization efficiency (Chew et al., 2018). The organic fertilizer processed by food waste and substrate in different ratios could promote the growth of potted vegetables pepper (*Capsicum annuum*) and cabbage (*Brassica pekinensis*) (Li et al., 2020). In addition, some investigators who directly applied food waste to potting soil found that food waste promoted leaf growth of *Chlorophytum comosum*, and increased soil available nitrogen, phosphorus and potassium (Song et al., 2014). Therefore, the ability of food waste in producing organic foods suggested here might be an alternative and effective way of treating biodegradable household garbage in the future.

Just recently, our team had proposed that biomass resources originally produced by photosynthesis and their biodegradable derivatives such as biodegradable household garbage, can be all used as organic fertilizers which have huge potentials in replacing chemical ones (Cui et al., 2021). Under the action of microorganism, the organic matter in the food waste will be rapidly decomposed into substances that can be easily absorbed by agriculture plants, thus partly replacing the usage of chemical fertilizers. In China, the actual amount of chemically synthesized nitrogen fertilizer absorbed by agricultural plants nationwide was 6.20 × 10^6^ t·a^−1^. Food waste, if applied as organic fertilizers, could replace 11% of chemical nitrogen application. Especially, the rural food waste when simply stacked, processed and returned directly to the farmland, could save processing and transportation costs. This will not only fully make use of household garbage, but also make up for the lack of organic fertilizers in the development of organic farming, so as to ensure food security.

### The relationship between GDP and food waste

Food waste occurs at all stages of the supply chain which is affected by many factors, such as geography and economy, production systems, infrastructure, markets, and consumption (Bonadonna et al., 2019). Larger consumer market and more consumption input undoubtedly exacerbate the food waste (Di Talia, Simeone & Scarpato, 2019).

Our study displayed that there was a very significant linear positive correlation between the per capital GDP and per capital food waste production (Fig. 5). GDP is mainly based on the classification of cities based on the concentration of commercial resources, diversity of lifestyles, future plasticity, urban hubs, and urban occupant activity index in China (Wang, 2010; Wang, 2018). Our findings demonstrated that the GDP and food waste generation of economically developed provinces were much higher than those of underdeveloped provinces (Fig. 5). And the largest amount of *per capita* biodegradable household garbage was in the sourthern China of the seven regions of the country (Table 3). This might be owing to its own economic conditions and geographical location. Southern China has been mostly economically developed, with the amount of waste generated remaining the highest over the years (Tian et al., 2018). This situation is consistent with that of the United States (Breunig et al., 2017). Food waste contains biomass nitrogen which could be used again for food production after simple treatment. If well paid, farmers might be actively mobilized and engaged in organic farming, so as to implement waste sorting and utilization at the source, reduce the load of rural waste entering to cities and cut down waste disposal costs.

### Questions and suggestions

So far, most investigations on food waste treatments usually focus on energy recovery. However, they do not consider the economic feasibility of such an approach (Ma & Liu, 2019). Food waste can be easily collected from various sources such as food processing industries, households, and hospitality sectors (Paritosh et al., 2017; Sindhu et al., 2019). Nevertheless, food waste may contain some inert materials, such as glass or plastic, and the distribution is somewhat difficult.

Although the prices of organic food are much higher and farmers are willing to invest their labors to use food waste in organic farming, correct collection, storage, and distribution are major obstacles of food waste management. To overcome those shortages, we here suggest: (1) The government should improve the household garbage collection and classification system in rural areas, and encourage farmers to use biodegradable household garbage in organic farming. By doing so, almost 80% of the degradable garbage in China could used as organic fertilizers to produce organic foods, thus uniting the ecological and economic chains together; (2) In cities, the government should encourage companies to build special food waste treatment plants to solve the problem of destination of urban household garbage by market-based means; (3) The government should supplement waste disposal subsidies to enterprises or farmers who produce organic food using the degraded food waste, if they have actually reduced the amount of biodegradable waste.

## Conclusions

The total amount of urban and rural biodegradable household garbage in China was 6.76 × 10^7^ t·a^−1^, with the nitrogen reservoir being 7.59 × 10^5^ t·a^−1^. The nitrogen reservoir of food waste potentially used as organic fertilizers reached to 6.53 × 10^5^ t·a^−1^, being equivalent to 11% of the amount of actual absorption for synthetic nitrogen fertilizers (6.20 × 10^6^ t·a^−1^) by agriculture plants of the country. There was a singificant correlation between per capital GDP and the per capital food waste. Food waste from household garbage should be classified and processed at the source, economically used as organic fertilizers to replace chemical ones. Our innovative solution might realize the recycling of biodegradable waste and the sustainable development of agriculture, ensure both food security and environmental protection.

## Additional Information and Declarations

### Data Availability

The following information was supplied regarding data availability: The raw measurements are available in the Supplemental File.
